# Intimate Partner Violence and Its Predictors among Pregnant Women in Eastern Ethiopia: Generalized Structural Equation Modeling

**DOI:** 10.1155/2022/7827234

**Published:** 2022-08-05

**Authors:** Tadesse Misgana, Adisu Birhanu Weldesenbet, Dawit Tamiru, Mandaras Tariku, Dejene Tesfaye, Daniel Alemu, Berhe Gebremichael, Merga Dheresa

**Affiliations:** ^1^Department of Psychiatry, College of Health and Medical Sciences, Haramaya University, Harar, Ethiopia; ^2^School of Public Health, College of Health and Medical Sciences, Haramaya University, Harar, Ethiopia; ^3^Department of Midwifery, College of Health and Medical Sciences, Haramaya University, Harar, Ethiopia; ^4^School of Nursing and Midwifery, College of Health and Medical Sciences, Haramaya University, Harar, Ethiopia

## Abstract

**Introduction:**

Intimate partner violence (IPV) has a negative impact on women's physical, mental, sexual, and reproductive health. Identifying the determinant factors of IPV among pregnant women is of paramount importance to overcome its negative consequences thereby increasing the performance of women in all activities. Thus, this study applied a generalized structural equation model (GSEM) to determine the prevalence of intimate partner violence among pregnant women and its predictors in Eastern Ethiopia.

**Methods:**

A community based cross-sectional study was conducted in Kersa Health and Demographic Surveillance System (KHDSS), Eastern Ethiopia. Data were collected form a sample of 1051 pregnant women using structured questionnaires. Descript findings were presented in percentage with 95% confidence interval. The generalized structural equation model was used to determine factors associated with each domain of IPV (physical, emotional, and social violence). Adjusted odds ratio (AOR) with a 95% CI were used to declare significant factors associated with intimate partner violence.

**Results:**

The overall prevalence of IPV in the Eastern Ethiopia was 48.57% (95% CI: 45.45, 51.69). The highest intimate partner violence was observed in the sexual domain of IPV (31.6%, 95% CI: (28.8, 34.58)). In GSEM, being a farmer (AOR = 0.42, 95% CI: 0.19, 0.91) was significantly associated with psychological domain of IPV. Age (AOR = 0.97, 95% CI: 0.95, 0.99) and educational status (neither read nor write) (AOR = 2.50, 95% CI: 1.61, 3.89) were significantly associated with physical domain of IPV. Being in medium (AOR = 0.64, 95% CI: 0.46, 0.90) and rich (AOR = 0.53, 95% CI: 0.36, 0.78), wealth quintiles were significantly associated with sexual domain of IPV, whereas husband controlling behavior was significantly associated with all domains of IPV.

**Conclusions:**

The magnitude of IPV among pregnant women was relatively high in Eastern Ethiopia. This finding pin a light to pay special consideration to pregnant women at each point of service delivery to alleviate consequence of IPV. Being a farmer, older ages and being in higher wealth quintiles were protective factor, whereas being uneducated increase the risk of IPV. Improving socioeconomic status and promoting legal rights of women is needed to alleviate the problem, and younger women require special attention.

## 1. Introduction

Intimate partner violence is the intentional use of physical force or power against a woman or man within a relationship which resulting in injury, psychological harm, and death. It is a major public health problem and a violation of women's human rights worldwide with negative impact on women's physical, mental, sexual, and reproductive health [1, 2].

Intimate partner violence during pregnancy is dangerous for both the mother and the fetus due to morbidity and mortality associated with violence and its long-term impact on women and indirectly children's mental and physical health [3, 4]. Intimate partner violence is a public health problem among women of reproductive age group. Women's victimized of physical and sexual violence by intimate partners has a risk of 50 to 70% to be affected by gynecological, central nervous system, and stress-related problems [5].

Worldwide around 49% of ever-married women faced physical violence, 59% of them experienced sexual violence, 71% of them had one or the other form of violence, or both, over their life time. About 35% of all ever-married women experienced at least one severe form of violence by a partner. About 47% of ever-married women in Kenya and one in four (21.5%) women of the reproductive age group in Nigeria faced IPV at some point in their lives [6, 7].

Ethiopia is among countries with the highest prevalence of IPV (71%). The prevalence of emotional or psychological violence was the highest (57.8%) followed by physical violence (32.2%) and sexual violence (7.6%) [6, 8]. Identifying the determinants of IPV among pregnant women is of paramount importance to overcome its negative consequences thereby to increase the performance of women in all activities. Despite this fact, there is a dearth of evidence on the magnitude of IPV and associated factors among rural pregnant women using the generalized structural equation model (GSEM). Although few studies were conducted on the topic, they did not consider identified associated factors across all domains of IPV and used binary logistic regression model which did not handle more than one outcome variable.

Thus, this study applied the generalized structural equation model (GSEM) to determine the prevalence of intimate partner violence among pregnant women and its predictors in Eastern Ethiopia. The use of GSEM enables us to handle more than one dependent variable (three domains of IPV). The finding pin a light on magnitude of IPV and its predictors among rural pregnant women, so as it helps health care providers and health programmer to solicit a tailored intervention.

## 2. Methods

### 2.1. Study Setting and Period

A community-based cross-sectional study was conducted in Kersa Health and Demographic Surveillance System (HDSS) in Kersa and Haramaya sites from January 30 to April 30, 2021. Kersa HDSS site is one of the 11 HDSS sites in the Ethiopian universities intended to reflect the countries' health and demography. Kersa Health Demographic Surveillance System was established in 2007. Kersa HDSS is located in Kersa District, Oromia Regional State, Eastern Ethiopia, and has a total population of around 50,830 of which 22.3% are women of child bearing age. The Kersa HDSS covers 24 of the 38 kebeles, with four health centers and ten health posts. Most inhabitants are farmers, with a minority working in small-scale trade, government posts, or casual laborers.

Haramaya Woreda (district) is located in the East Hararghe Zone of Oromia Regional State. The capital town, Haramaya, is located about 506 km from Addis Ababa. Haramaya Woreda has thirty-three rural and two urban kebeles (subdistricts) with a total population of 220,986 of which 23.86% were women of reproductive age group women. In the district, one district hospital, seven health centers and 34 health posts are providing health service. In addition, Haramaya HDSS covers 12 rural kebeles [9, 10].

### 2.2. Study Design and Population

A community-based cross-sectional study was conducted. All pregnant women who were on follow-up at Kersa HDSS were the study population.

### 2.3. Sample and Sampling Procedure

The kebeles were stratified in to urban and rural. Then, 10 out of 24 rural kebeles and 9 out of 19 urban kebeles were selected using the lottery method. The total sample size was proportionally allocated to each stratum, and all study participants from the selected kebeles were included. From selected kebeles, all 1015 pregnant women satisfying the inclusion criteria were included in the study.

### 2.4. Data Collection Tool and Procedures

Structured, interviewer administered questionnaire was used to collect data on sociodemographic, economic characteristics, substance use, and intimate partner violence. The questionnaire has five items for psychological violence, five items for physical violence, and three items for sexual violence. Participants who respond “Yes” to one or more items of violence during recent pregnancy were considered as victims of intimate partner violence. The data were collected by face-to-face interview using structured and semistructured questionnaires prepared in Open Data Kit (ODK) collect form, and completed data were sent directly to the server. Pregnant women were interviewed in separate rooms in their home environment or inside their compounds during working days of the week.

### 2.5. Operational Definitions

#### 2.5.1. Intimate Partner Violence

Women reported that they experienced any act of physical, sexual, or emotional (psychological) violence or any combination of the three by an intimate partner during current pregnancy.

#### 2.5.2. Psychological Violence

Women reported that they experienced one or more acts such as insults, humiliation, scare or intimidation, and threatened when asking their friends/family and threatened to hurt by an intimate partner during the current pregnancy.

#### 2.5.3. Physical Violence

Women experienced one or more acts such as slapped, pushed or shoved, kicked, hit their abdomen during pregnancy, strangled, and threatened to use or actually used a gun, knife, or other weapon by an intimate partner during current pregnancy.

#### 2.5.4. Sexual Violence

Women reported that they experienced one or more acts or threats such as forced into sexual intercourse when she did not want, had sexual intercourse when she did not want to because she was afraid of what partner might do, and forced to do something sexual that she found degrading or humiliating by an intimate partner during current pregnancy.

### 2.6. Data Processing, Model Building, and Analysis

After collection, data was edited and cleaned; each questionnaire was checked for completeness and coded. Data was entered into computer using EpiData version 3.1, and the analysis was done using STATA version 14.0. Categorical variable was described using frequencies and percentages. The generalized structural equation model (GSEM) was used to determine factors associated with each domain of IPV (physical, emotional, and social violence). Each domain of IPV was binary variable that was analyzed with binomial family and a logit link function. Husband controlling behavior was a latent variable which constitutes items with yes/no response, and their measurement model was analyzed with the binomial family with logit link function. Statistically significant effects were assumed for *P* value <0.05 at confidence interval of 95%.

### 2.7. Ethical Consideration

Ethical clearance was obtained from Haramaya University College of Health and Medical Sciences Institutional Health Research Ethics Review Committee (IHRERC) and was submitted to the Woreda health department of each study site. The objectives and purposes of the studies as well as risks and benefits according to the level of understanding of participants were provided. Confidentiality of the data was strictly followed and in order to keep privacy, participants was interviewed in separate rooms in their home environment or inside their compounds in the manner their privacy is maintained in the community. The informed written and signed consent was obtained from the participants.

## 3. Results

### 3.1. Descriptive Characteristics of Pregnant Women

A total of 1015 pregnant women fulfilling inclusion criteria were included in the final analysis. The mean age of pregnant women that participated in this study was 30.12 (±8.47) years. More than three fourth 772 (76.06%) of study participant cannot read and write, and 900 (88.66%) were housewife. With regard to marital status 982 (96.76%) of pregnant women were married and concerning wealth quintiles, majority 388 (38.23%) were in poor wealth quintiles (Table 1).

### 3.2. Prevalence of IPV among Women

**Figure 1:**
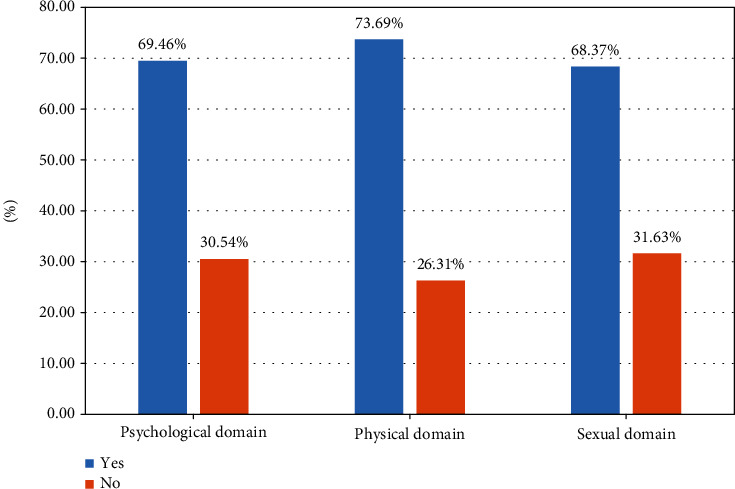
Prevalence of intimate partner violence across three domains of IPV among pregnant women in Eastern Ethiopia, 2021.

The overall prevalence of all form of IPV in the study area was found to be 48.57% (95% CI: 45.45, 51.69). The highest intimate partner violence was observed in the sexual domain of IPV (31.6%) with 95% CI: (28.8, 34.58) and the lowest in physical domain of IPV (26.3%) with 95% CI: (23.6, 29.1). The prevalence of IPV in psychological domain was 30.54% (95% CI: 27.72, 33.48) (Figure 1).

Among 321 pregnant women who were victims of sexual violence, 23.15% of them were physically forced to have sex when they did not want, 16.35% of them have sexual intercourse while they are worried having sex during pregnancy, and 11.53% of them were ever forced sexually in a way they did not approved.

Out of 267 physically abused pregnant women, slapping (15.27%) and pushing/shoving (11.03%) were the majorly reported by respondents. Regarding psychological violence, the majority of victimized pregnant women 16.65% were insulted or made feel bad, and 11.82% reported being scared or intimidated on purpose by intimate partner **(**Table 2**).**

### 3.3. Determinants of Intimate Partner Violence

**Figure 2:**
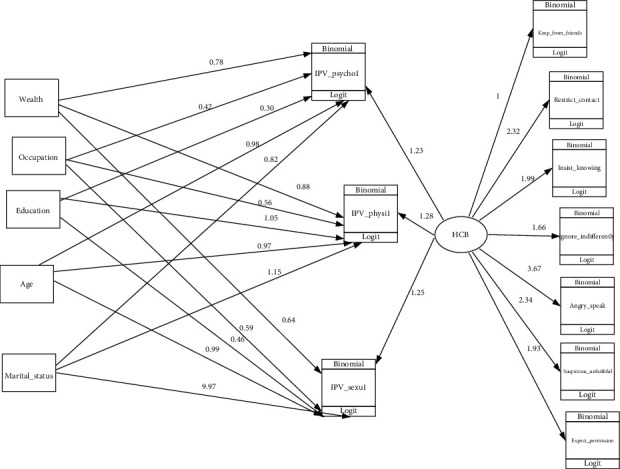
GSEM for determinant factors of intimate partner violence among reproductive age group women in Eastern Ethiopia, 2021.

As shown in Figure 2 the final model has included six exogenous variables of which one is latent variable (husband controlling behavior) and five are observed variables (age, marital status, educational status, occupational status, wealth index) and ten endogenous (physical, emotional, and sexual violence) and seven indicator variables that measure husband controlling behavior. Occupational status was significantly associated with psychological domain of IPV. Age and husband controlling behavior were variables significantly associated with physical domain of IPV. Wealth index and husband controlling behavior were significantly associated with sexual domain of IPV (Table 3).

The likelihood of experiencing psychological violence was decreased by 58% among pregnant women who are farmer as compared to those who are privately employed (AOR = 0.42, 95% CI: 0.19, 0.91) keeping other variables in the model constant.

For one year increase in age, the odds of physical violence among pregnant women was decreased by 3% (AOR = 0.97, 95% CI: 0.95, 0.99) adjusting for other variables. The likelihood of experiencing physical violence was 2.5 times higher among pregnant women who cannot read and write (AOR = 2.50, 95% CI: 1.61, 3.89) as compared to those who are literate. Substance use was associated with 45% decrease in physical violence among pregnant women (AOR = 0.55, 95% CI: 0.36, 0.84).

The odd of experiencing sexual violence was decreased by 36% and 47% among women in middle quintiles (AOR = 0.64, 95% CI: 0.46, 0.90) and rich wealth quintiles (AOR = 0.53, 95% CI: 0.36, 0.78) as compared to those in poor wealth quintiles. The latent variable husband controlling behavior was significantly associated with all domain of IPV (psychological, physical, and sexual (Table 3)).

## 4. Discussion

The overall prevalence of IPV during pregnancy was 48.57% (95% CI: 45.45, 51.69). This result has a great implication on maternal and child health. About and half of pregnant women suffer with IPV, and it implies that pregnant women would suffer with immediate and long-term psycho social complication. This finding is in line with previous studies from Uganda (48%) but higher than prevalence of IPV reported from Oromia (35.6% [11] and Tigray region of Ethiopia 37.5% [12]). The possible explanation for discrepancy in prevalence of IPV in the current study compared to previous study was the difference in study design. The previous studies in Ethiopia were institutional-based and might miss cases of IPV in the community, whereas the current study was community based. The difference might also be due to difference in study setting as the current study was conducted in Eastern part of Ethiopia where the prevalence of substance use by intimate partner was estimated to be very high [13, 14]. In addition, the involvement of women in economic activities might be considered as a challenge in power sharing with man hence acted more provocatively [15].

Regarding magnitude of IPV across domains of IPV, the 31.6% had sexual violence which is in line with worldwide sexual violence estimate by WHO [16]. Psychological and physical violence was experienced by 30.5% and 26.3% of pregnant women, respectively, and this finding is lower than 57.8% that reported from Gondar, Ethiopia [8]. The difference might be due to difference in tool used to assess IPV. The study in Gondar used four-item WHO tool, whereas this study used five-item tools to assess magnitude of IPV [17].

Occupational status was significantly associated with psychological domain of IPV. Pregnant women who are farmers were less likely to have psychological violence (AOR = 0.42, 95% CI: 0.19, 0.91) compared to privately employed women, and this could be due to underreporting of sexual abuse by women who are farmers compared to employed women due to the sensitivity of the issue and the surrounding cultural taboos as farmers are primarily from rural areas.

Increase in women's age was associated with lower odds of experiencing physical violence (AOR = 0.97, 95% CI: 0.95, 0.99). This might be higher access to information on legal rights of women among women at higher age.

Higher wealth index quintiles were associated with decrement in sexual violence among pregnant women, and this might be explained by low socioeconomic status lead to aggressive behavior seen among most intimate partners.

Pregnant women who cannot read and write had 2.5 times higher odds of experiencing physical violence compared to literate women (AOR = 2.50, 95% CI: 1.61, 3.89). This finding is consistent with study conducted in Ethiopia, Nigeria, and Tanzania [18–20], and this might be due to higher access to information concerning women empowerment among literate women. Uneducated women are more likely to accept IPV compared to literate women, and this could be another possible explanation for the finding.

Consistent with the study from Ghana [21] and Gambia [22], husband controlling behavior was significantly associated with IPV among pregnant women. This could be due to community perception on male superiority.

### 4.1. Strength and Limitation

The good side of this study is the use of validated instruments for assessing magnitude of IPV.

This study also has some important limitations that should be considered when interpreting the results. The study might be affected by social desirability bias since study participant may not be volunteer to disclose their violence.

### 4.2. Conclusion and Recommendations

The magnitude of IPV among pregnant women was relatively high in Eastern Ethiopia. This finding pin a light to pay special consideration to pregnant women at each point of service delivery to alleviate consequence of IPV. Being a farmer, older ages and being in higher wealth quinti were protective factor, whereas being uneducated increases the risk of IPV. Improving socioeconomic status and promoting legal rights of women are needed to alleviate the problem, and younger women requires special attention.

## Figures and Tables

**Table 1:** Descriptive characteristics of pregnant women in Eastern Ethiopia, 2021 (*N* = 1015).

Variables	Category	Frequency	Percent%
Age	Mean (±SD)	30.12 (8.47)	
Religion	Muslim	1012	99.70
	Orthodox	3	0.30
Educational status	Literate	218	21.48
	Read only	4	0.39
	Can read and write	21	2.07
	Cannot read and write	772	76.06
Occupational status	Farmer	47	4.63
	Housewife	900	88.67
	Student	23	2.27
	Unemployed	31	3.05
	Other	14	1.38
Marital status	Married	982	96.75
	Widowed/divorced	10	0.98
	Living together	23	2.27
Wealth quintiles	Poor	388	38.23
	Middle	375	36.95
	Rich	252	24.83

**Table 2:** Prevalence of intimate partner violence among pregnant women in Eastern Ethiopia, 2021 (*n* = 1015).

Violence item	Frequency	Percent
Psychological violence		
Insulted you or made you feel bad about yourself?	169	16.65
Belittled or humiliated you in front of other people	79	7.78
Done things to scare or intimidate you on purpose (e.g., the way he looked at you, by yelling)	120	11.82
Threatened when asking your friends/family	88	8.67
Threatened to hurt you or someone you care about	94	9.26
Physical violence		
Slapped you or threw something at you that could hurt you?	155	15.27
Push you or shoved you?	112	11.03
Kicked, dragged you, or beaten you up	109	10.74
During pregnancy, hit your abdomen with a fist or with something else	94	9.26
Strangled, choked, or burnt you on purpose?	82	8.08
Threatened to use or actually used a gun, knife, or other weapon against you?	77	7.59
Sexual violence		
Physically forced you to have sexual intercourse when you did not want to?	235	23.15
Did you ever have sexual intercourse you did not want because you were afraid of what might be done to you?	166	16.35
Did anyone ever forced you sexually in a way you did not approved?	117	11.53

**Table 3:** Determinant factors of intimate partner violence among reproductive age group women in Ethiopia, 2021.

Variables	Category	Psychological violence		Physical violence		Sexual violence	
		AOR	95% CI	AOR	95% CI	AOR	95% CI
Age	—	0.98	0.96, 1.01	0.97	0.95, 0.99	0.99	0.98, 1.01
Occupational status	Farmer	0.42	0.19, 0.91	0.56	0.26, 1.22	0.59	0.29, 1.21
	Privately employed	Ref.	Ref.	Ref.	Ref.	Ref.	Ref.
	Unemployed	0.80	0.33, 1.99	0.63	0.24, 1.66	0.83	0.34, 2.03
	Other	0.76	0.20, 12.80	0.95	0.25, 3.63	0.77	0.21, 2.85
Marital status	Married	Ref.	Ref.	Ref.	Ref.	Ref.	Ref.
	Separated/divorced	0.82	0.07, 9.23	1.15	0.09, 13.35	9.97	0.79, 125.19
	Widowed	3.89	0.59, 25.73	0.75	0.22, 14.01	3.05	0.46, 20.24
	Living together	0.67	0.20, 2.22	1.20	0.37, 3.89	1.53	0.54, 4.33
Educational status	Literate	Ref	Ref	Ref	Ref	Ref	Ref
	Read or write	0.30	0.08, 1.12	1.05	0.33, 3.28	0.46	0.15, 1.42
	Cannot read and write	1.40	0.96, 2.06	2.50	1.61, 3.89	1.22	0.84, 1.79
Wealth quintiles	Poor	Ref.	Ref.	Ref.	Ref.	Ref.	Ref.
	Middle	0.78	0.55, 1.09	0.88	0.61, 1.27	0.64	0.46, 0.90
	Rich	0.88	0.59, 1.29	0.68	0.44, 1.03	0.53	0.36, 0.78
HCB		1.23	1.14, 1.32	1.28	1.17, 1.40	1.25	1.15, 1.34

HCB: husband controlling behavior; AOR: adjusted odds ratio; COR: crude odds ratio; IPV_psycho1: psychological domain of intimate partner violence; IPV_physi1: physical domain of intimate partner violence; IPV_Sexu1: sexual domain of intimate partner violence; keep_from_friends: try to keep you from seeing your friends; restrict_contact: tries to restrict contact with the family of birth; insist_knowing: insist on knowing where you are all time; ignore_indifferently: ignore you and treat you indifferently; angry_speak: get angry if you speak with another man; Suspicious_unfaithful: is often suspicious that you are unfaithful; Expect_permission: expect you to ask for permission before you seeking health care for yourself.

## Data Availability

All necessary information were included within the manuscript.

## Abbreviations

AOR: Adjusted odds ratio
CI: Confidence interval
COR: Crude odds ratio
GSEM: Generalized structural equation model
HIV: Human immune deficiency virus
IHRERC: Institutional Health Research Ethics Review Committee
IPV: Intimate partner violence
KHDSS: Kersa Health and Demographic Surveillance Site
SD: Standard deviation
WHO: World Health Organization.
